# Micropatterned, clickable culture substrates enable *in situ* spatiotemporal control of human PSC-derived neural tissue morphology†
†Electronic supplementary information (ESI) available. See DOI: 10.1039/c4cc08665a



**DOI:** 10.1039/c4cc08665a

**Published:** 2015-02-17

**Authors:** G. T. Knight, J. Sha, R. S. Ashton

**Affiliations:** a Department of Biomedical Engineering , University of Wisconsin , Madison , WI 53706 , USA . Email: rashton2@wisc.edu; b BIONATES Theme , Wisconsin Institute for Discovery , Madison , WI 53715 , USA; c School of Mechanical and Power Engineering , East China University of Science and Technology , Shanghai , China

## Abstract

We describe a modular culture platform that enables spatiotemporal control of the morphology of 2D neural tissues derived from human pluripotent stem cells (hPSCs) by simply adding clickable peptides to the media. It should be widely applicable for elucidating how spatiotemporal changes in morphology and substrate biochemistry regulate tissue morphogenesis.

## Abstract 2

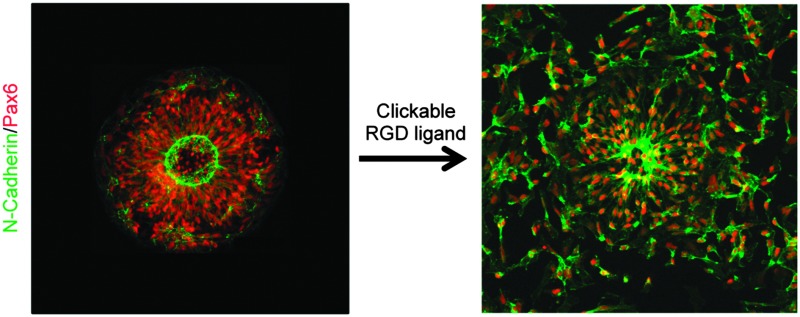
 *In situ* regulation of the morphology of neural tissues derived from human pluripotent stem cells using micropatterned, clickable substrates.

When differentiated as high density 2- and 3-D aggregates, human pluripotent stem cells (hPSCs), *i.e.* human embryonic and induced pluripotent stem cells, can spontaneously form tissues that recapitulate early stages of developmental morphogenesis. In 2-D culture, this was first observed during neural differentiation of hPSCs,^1^ but the seemingly limitless potential of hPSCs in *in vitro* morphogenesis was only recently realized with the derivation of 3-D brain, retinal, intestinal, and kidney organoids.^2^ Such tissues contain microscale structures with architectures mimetic of respective developing human tissues. However, orchestration of the morphogenesis process at the macroscale is chaotic, generating tissues with unnatural morphologies and anatomy.^3^ To fully harness the capabilities of hPSCs and advance towards reproducible engineering of organoids with biomimetic anatomy, culture platforms that enable facile, *in situ* spatiotemporal control of hPSC-derived tissue morphology and cellular differentiation must be developed.

Current methods for inducing *in situ* spatiotemporal changes in tissue morphology *in vitro* require perturbations of ideal culture conditions such as temperature,^4^ pH,^5^ UV light exposure,^6^ and solvent concentrations^7^ or physical destruction of parts of the tissue or culture substrate. Additionally, approaches using lasers^8^ or electrochemistry^9^ to actuate *in situ* reactions require complex integration of the culture system with specialized equipment thereby limiting their usage. To avoid such complications, we developed a culture platform in which coverslip substrates are engineered *a priori* with micropatterned PEG brushes presenting azide groups that can undergo copper-free click reactions with modular peptide–DBCO conjugates.^10,11^ Upon media supplementation, the peptide conjugates are readily immobilized onto the culture substrates in a spatiotemporal and quantitative manner (Scheme 1). Using clickable conjugates with biomimetic fibronectin peptide sequences, we demonstrated *in situ* conversion of inert PEG brushes, which initially confined hPSC-derived neural tissues to a microscale circular morphology, into biospecific, cell-adhesive substrates that permitted radial tissue growth. This progression in tissue morphology mimics early morphogenesis of the developing central nervous system (Fig. S1A, ESI†), and generated arrays of tissues with architecture analogous to developing neural tube slice cultures. Therefore, our methodology for actuating spatiotemporal changes in the morphology of 2-D hPSC-derived tissues should be widely applicable given its compatibility with standard culture practices.

**Scheme 1:**
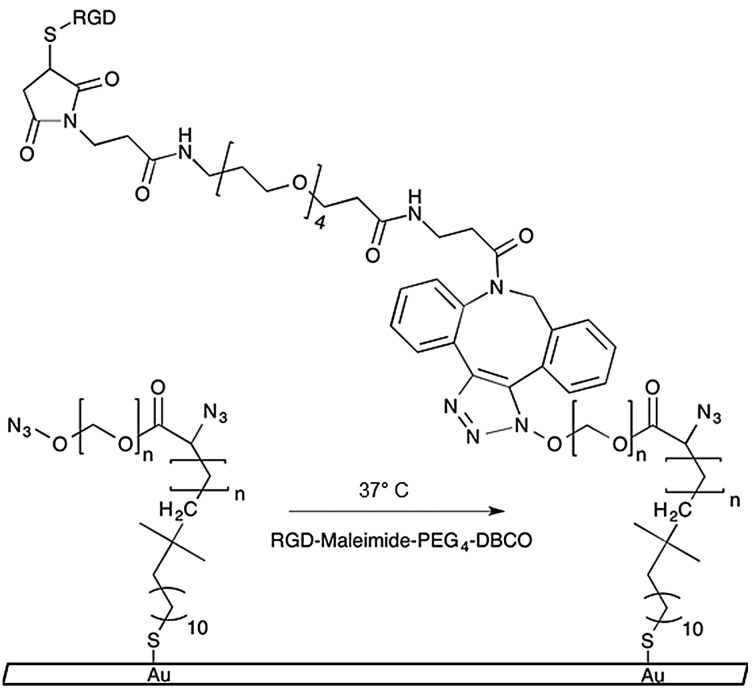
Azide-functionalized PEGMA-grafted substrates undergo 1,3-dipolar cycloaddition reaction with DBCO conjugated RGD–peptides.

We previously published a detailed synthesis protocol and characterization of our micropatterned culture substrates.^10^ In brief, the alkanethiol atom-transfer radical-polymerization (ATRP) initiator, ω-mercaptoundecyl bromoisobutyrate, was microcontact printed on gold-coated microscope slides.^12,13^ Then, surface-initiated activators generated by electron transfer (SI-AGET) ATRP of poly(ethylene glycol) methacrylate (PEGMA) was performed for 16 h resulting in PEGMA brushes grafted to the micropatterned regions. Next, a 4 h Steglich esterification reaction was performed to substitute the PEGMA side chains' hydroxyl groups with bromine, which served as leaving groups during a subsequent nucleophilic substitution with sodium azide. This produced micropatterned culture substrates decorated with PEGMA brushes densely presenting azide groups that can undergo strain-induced 1,3-dipolar cycloaddition “click” reactions with high strain molecules such as dibenzocyclooctyne (DBCO) to yield 1,4-substituted triazoles (Scheme 1).^10,11,14^

To synthesize cell-adhesive, clickable peptide conjugates, FITC-labelled RGD peptides (FITC-GPCGYGRGDSPK), containing a fibronectin integrin-binding motif and a cysteine residue,^15^ were conjugated to DBCO–PEG_4_–Maleimide linkers *via* Michael-type addition using a 4 : 1 molar excess of DBCO–PEG_4_–Maleimide. The fluorescent RGD peptide–DBCO conjugates (RGD–DBCO) were isolated using size exclusion chromatography and UV-Vis spectroscopy based on 309 and 492 nm peaks characteristic of DBCO and FITC, respectively (Fig. S2, ESI†). To assess whether RGD–DBCO spontaneously clicked onto micropatterned PEGMA-azide brushes under normal culture conditions, substrates presenting arrays of circular PEGMA-azide brushes 300 μm in diameter were fabricated. The slides were also backfilled with ω-mercaptoundecyl bromoisobutyrate to graft poly(ethylene glycol) methyl ether methacrylate (PEGMEMA) *via* the same SI-AGET ATRP protocol to render the remainder of the substrate non-fouling. Next, the micropatterned substrates were placed in 6-well plates, incubated in Essential 6 culture media (E6, Life Technologies) containing various concentrations of RGD–DBCO for 24 h at 37 °C, rinsed with water, and dried for analysis. To create a standard curve for RGD–DBCO surface density quantification, 0.5 μL droplets from serial dilutions of the stock RGD–DBCO solution were dried on top of non-micropatterned PEGMA substrates. Using confocal microscopy, the integrated flouresence intensity per area was calculated for multiple micropatterned PEGMA-azide regions on each experimental substrate and dried droplet areas on the standard curve substrates (Fig. 1 and Fig. S3, ESI†). Estimating from the standard curve, the achievable surface density of immobilized RGD–DBCO on PEGMA-azide brushes could be predictably varied between 0–55 pmol cm^–2^ by altering the media's RGD–DBCO concentration.

**Fig. 1:**
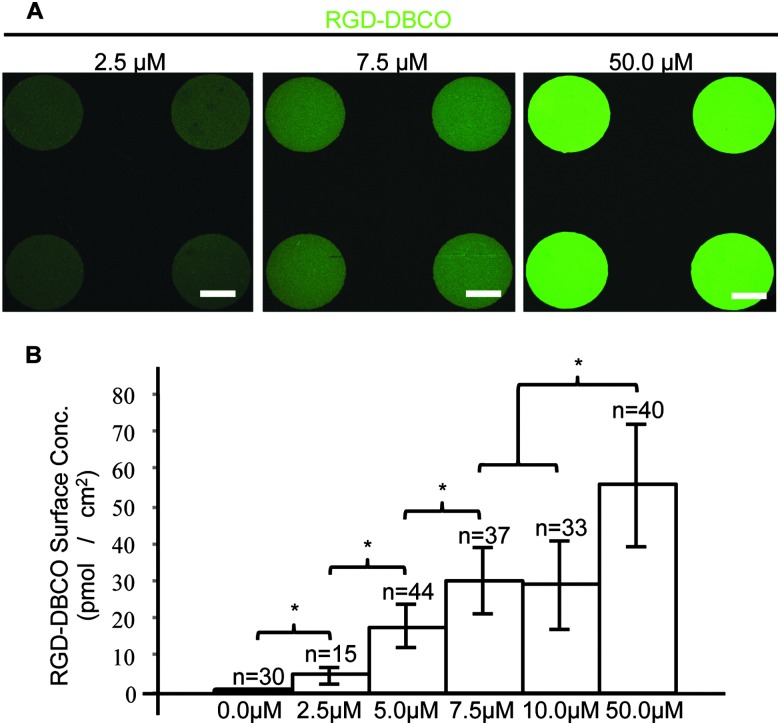
(A) Fluorescent images of micropatterned PEGMA brushes conjugated with RGD–DBCO at varying reaction concentrations, 150 μm scale bars. (B) Surface density of RGD–DBCO on micropatterned substrates across all reaction concentrations, *p < 0.05.

To test our culture system's ability to actuate tissue morphology changes in an *in situ* spatiotemporal manner, micropatterned substrates were fabricated with PEGMA-azide brushes everywhere except for an array of 300 or 900 μm diameter circles (Fig. 2A). The substrates were coated with 0.083 mg mL^–1^ matrigel in E6 media overnight at 37 °C and seeded with neural stem cells (NSCs) derived from WA09 hPSCs, as described and characterized in detail elsewhere,^16^ at 50 000 cells per cm^2^ in E6 media with 10 μM ROCK Inhibitor (Y-27632). The NSCs adhered only within the circular non-grafted regions, and after 2 days of culture generated similarly shaped tissues confined by the surrounding inert PEGMA-azide brushes (Fig. 2B). Each arrayed tissue consisted of polarized Pax6^+^/N-cadherin^+^ NSCs, and some tissues of ∼300 μm diameter even contained a single ring of polarized NSCs mimetic of the developing neural tube^17^ (Fig. 2C, Fig. S1A, ESI†). Then, we supplemented the culture media with either 0 (control) or 5 μM RGD–DBCO or 5 μM RDG-DBCO, which is not cell-adhesive. After 24 and 48 h of additional culture, progressive radial expansion of the arrayed neural tissues was only observed on substrates exposed to RGD–DBCO, indicating that *in situ* immobilization of peptide–DBCO conjugates on PEGMA-azide brushes created biospecific substrates (Fig. 2B). Similar *in situ* spatial changes in the morphology of ∼900 μm diameter neural tissues were observed after temporal addition of RGD–DBCO (10 μM) to the culture media. At both 24 and 96 h post-click functionalization of the culture substrates, the arrayed neural tissues continued expanding radially with a tissue architecture consisting of a central polarized NSC core producing outwardly migrating progeny (Fig. 3A and B, Fig. S1B, ESI†). Such architecture is analogous to slice cultures of the developing neural tube^17^ (Fig. S1A, ESI†), and it is uniquely generated in a high throughput arrayed fashion using the spatiotemporal control afforded by micropatterned, clickable culture substrates.

**Fig. 2:**
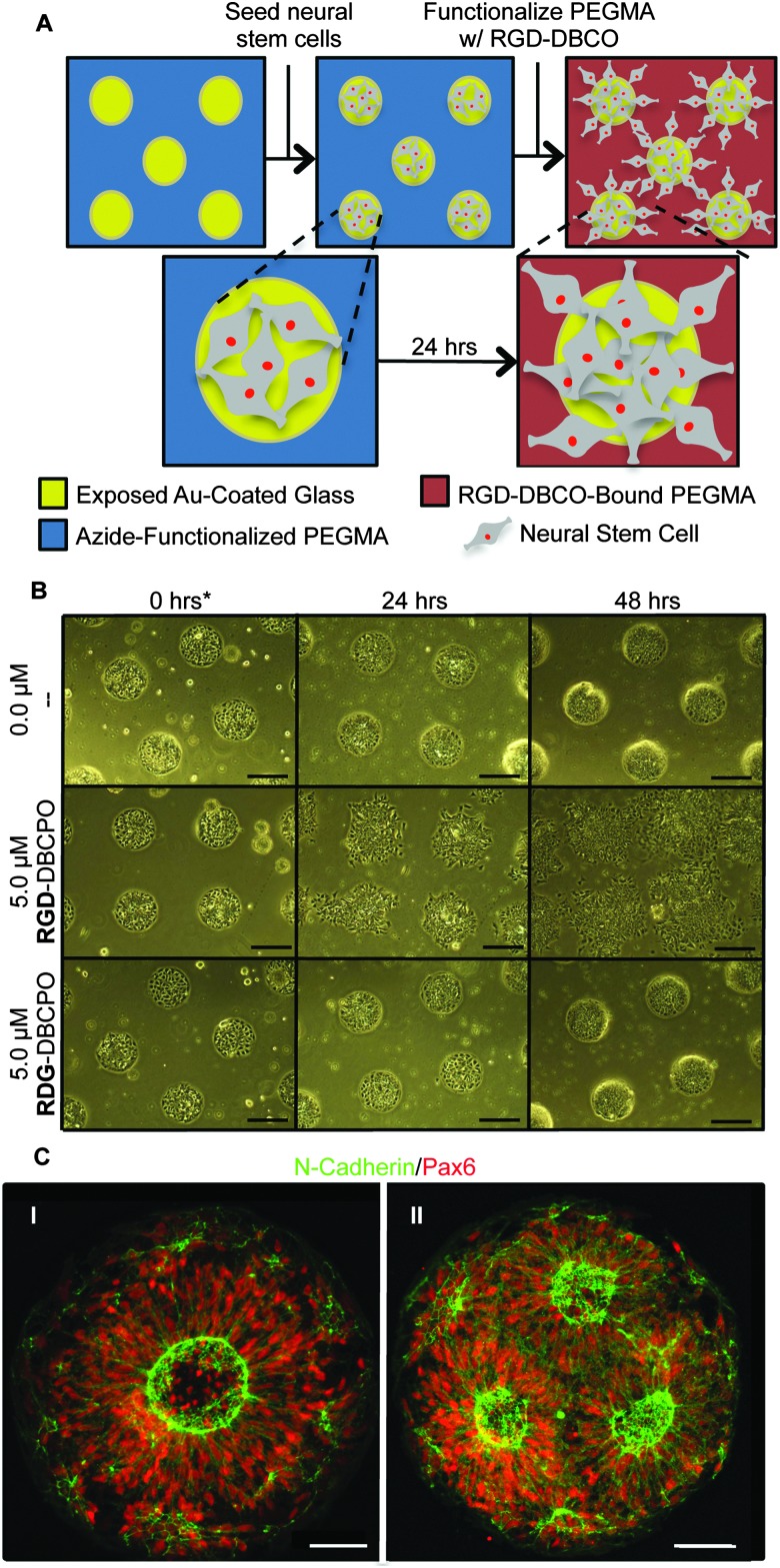
(A) Schematic of neural tissue outgrowth onto RGD–DBCO-modified micropatterend substrates. (B) Bright field images of neural tissue outgrowth onto PEGMA brushes following addition of RGD–DBCO at time = 0, 300 μm scale bars. (C) Fluorescent images of polarized NSCs on micropatterned substrates, 50 μm scale bars.

**Fig. 3:**
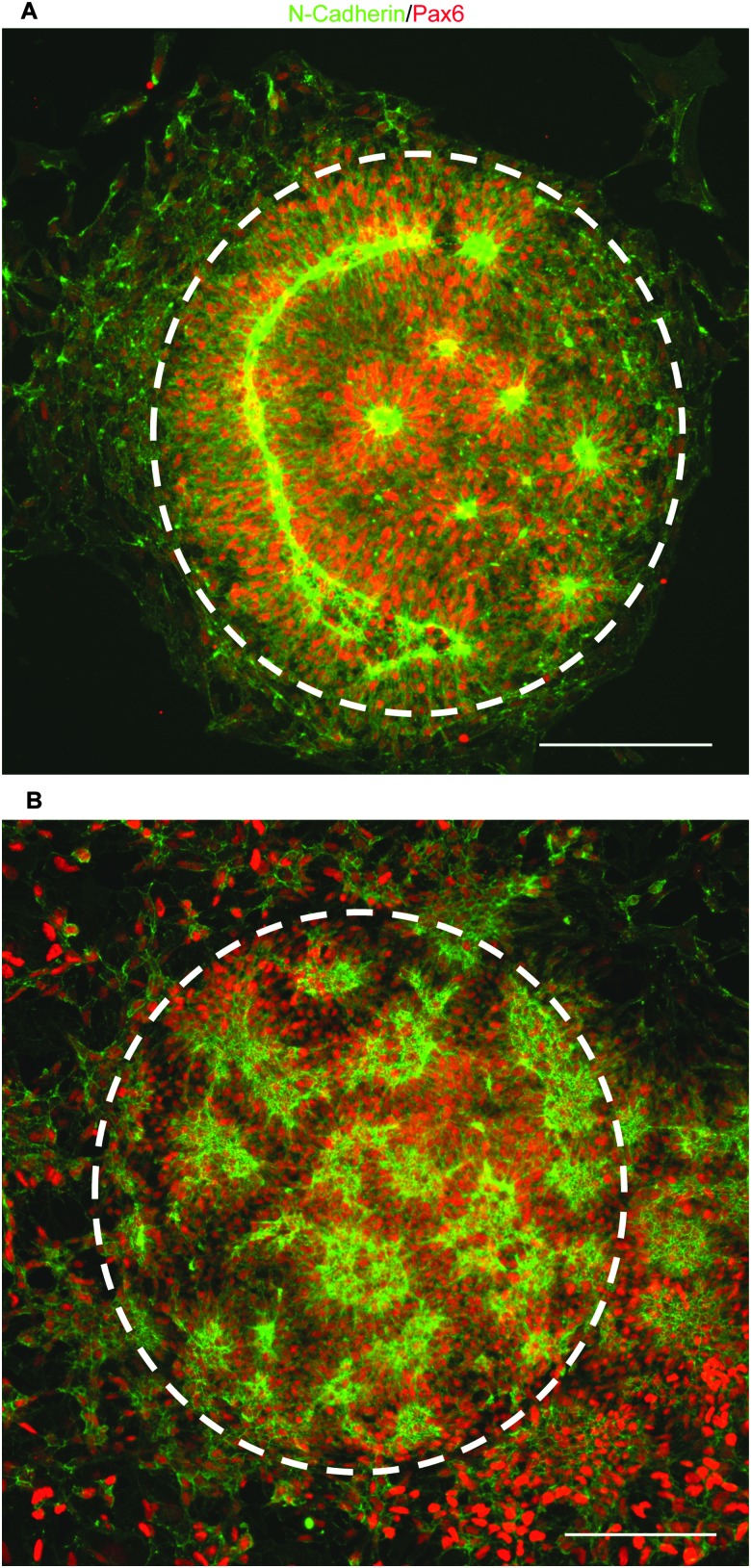
(A) Fluorescent images of neural tissues with a polarized NSC core and radial expansion due to migrating progeny at 24 (B) and 96 h after supplementation with 10.0 μM RGD–DBCO, 250 μm scale bars.

The generation of truly biomimetic tissues by harnessing *in vitro* morphogenesis of hPSCs will only be possible using culture platforms that enable spatiotemporal control of tissue morphology and cellular differentiation. Here, we described a culture platform based on micropatterned, clickable culture substrates that permits facile alteration of substrate biochemistry in a chemically defined, *in situ*, and spatiotemporal manner to dynamically regulate the morphology of hPSC-derived neural tissues. Additionally, the culture substrates could be engineered with multicomponent PEG brushes using robotic microcontact printing,^18^ which could be used to not only actuate a change in tissue morphology, but also confine tissues to a second pre-determined morphology. Given the variety of bioorthogonal and biocompatible “click” chemistry motifs available,^19^ the substrates could also be engineered to present multiple biological ligands, each in a discrete, physiologically relevant, microscale spatial orientation.^20^ Plus, the biospecific cell-ligand interactions enabled by peptide conjugated PEG brushes will facilitate reductionistic experimentation to elucidate the effects of pertinent biological cues on cell fate.^10,21^ Thus, micropatterned clickable substrates provide a highly modular culture platform for investigating how spatiotemporal changes in morphology and substrate biochemistry can be used to control *in vitro* morphogenesis of hPSC-derived tissue. Due to its compatibility with standard culture techniques, this approach should be broadly applicable in advancing our ability to generate hPSC-derived tissues *in vitro* with biomimetic anatomy.

This work was supported by the Wisconsin Institute for Discovery, the NINDS (1R21NS082618-01A1), and with Assistance Agreement No. 83573701 awarded by the EPA to R.S.A. It has not been formally reviewed by the EPA. The views expressed in this document are solely those of R.S.A. and do not necessarily reflect those of the Agency. The EPA does not endorse any products or commercial services mentioned in this publication. Also, R.S.A. holds an Innovation in Regulatory Science Award from the Burroughs Wellcome Fund.
