# Supplementary material for: Micropatterned, clickable culture substrates enable in situ spatiotemporal control of human PSC-derived neural tissue morphology
Source: Chem Commun (Camb). 2015 Feb 17;51(25):5238–41. doi: 10.1039/c4cc08665a (PMC4456773; doi:10.1039/c4cc08665a)
Supplement: Supplementary file 1 [file CC-051-C4CC08665A-s001.pdf]

Electronic Supplementary Material (ESI) for ChemComm.
This journal is © The Royal Society of Chemistry 2015

Micropatterned, clickable culture substrates enable *in situ* spatiotemporal control of human PSC-derived neural tissue morphology

G. T. Knight, J. Sha, and R. S. Ashton

Supplemental Information:

Supplemental Figure 1: relevant to Figure 2 and 3

Supplemental Figure 2: relevant to Figure 1

Supplemental Figure 3: relevant to Figure 1

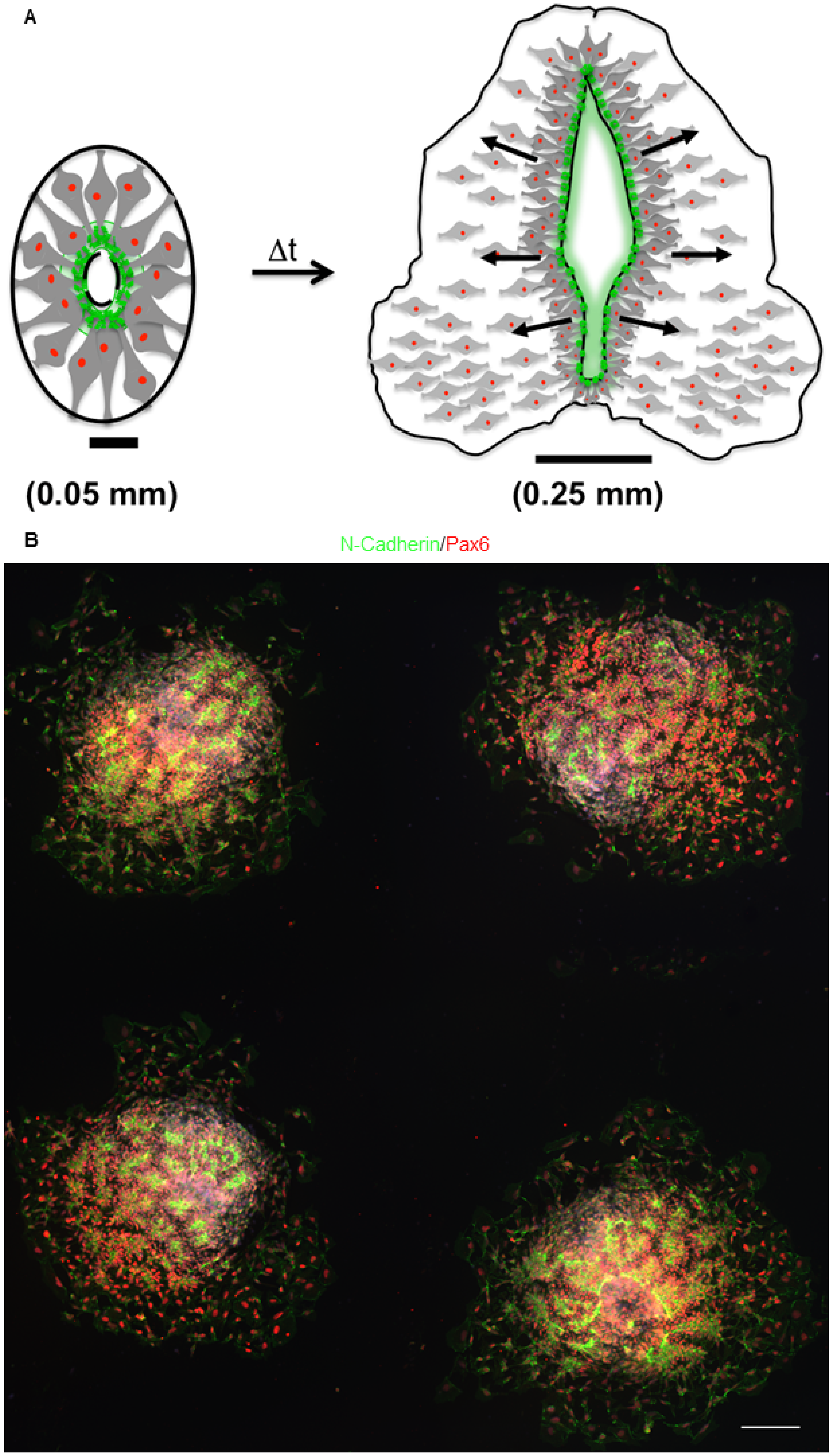

Supplemental Figure 1: A) Schematic of neural tube morphogenesis in early human development depicted as transverse slices. **B)** Fluorescent images of neural tissues with polarized NSC cores and radially expanding morphologies due to outgrowth of progeny at 24 hours post-click functionalization, 250 µm scale bars.

A

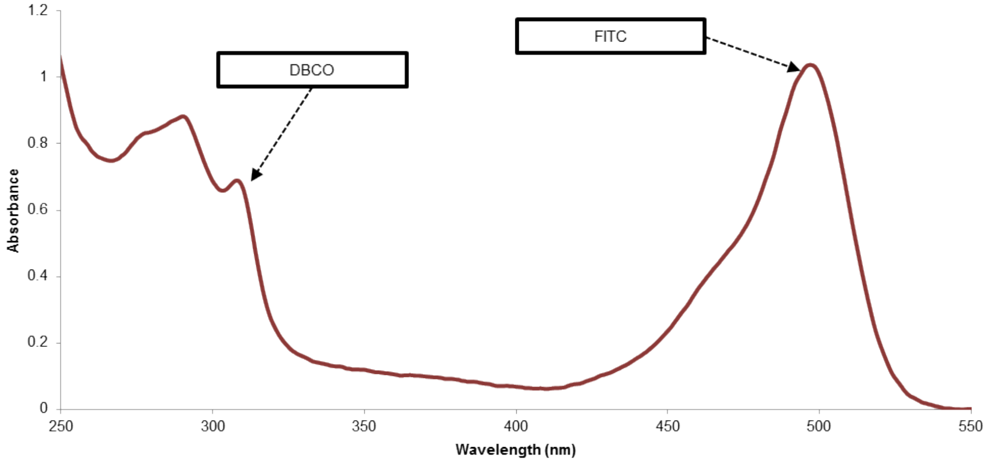

Supplemental Figure 2: A) UV-Vis spectra of FITC-RGD-DBCO with characteristic peaks at ~309 nm and ~492 nm denoting the presence of DBCO and FITC molecules, respectively.

A

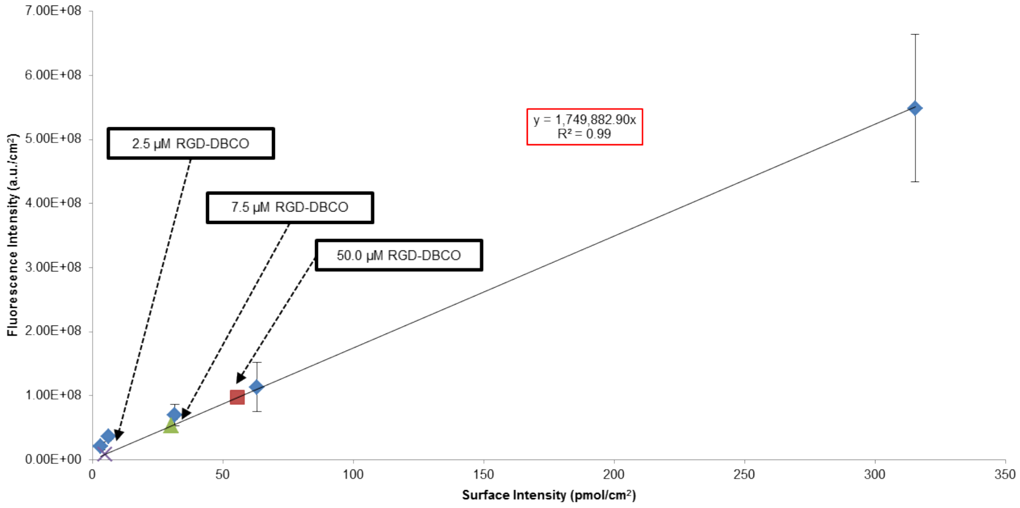

Supplemental Figure 3: A) Standard curve of FITC-RGD-DBCO with data points corresponding to concentrations of immobilized RGD-DBCO on functionalized PEGMA substrates.
